# A Qualitative Study on the Burden of Nonadherence to Antiretroviral Therapy Among People Living With HIV/AIDS in Jharkhand, India

**DOI:** 10.7759/cureus.73636

**Published:** 2024-11-13

**Authors:** Mithilesh Kumar, Neha Priya, Abhay Kumar, Anit Kujur, Atul Kachhap, Vidya Sagar, Ajit K Karn, Smiti Narain

**Affiliations:** 1 Community Medicine, Rajendra Institute of Medical Sciences, Ranchi, IND; 2 General Medicine, Rajendra Institute of Medical Sciences, Ranchi, IND; 3 Preventive Medicine, Rajendra Institute of Medical Sciences, Ranchi, IND; 4 Preventive and Social Medicine, Rajendra Institute of Medical Sciences, Ranchi, IND

**Keywords:** antiretroviral therapy, nonadherence causes, plhiv, qualitative studies, tribal

## Abstract

Background: Adherence to antiretroviral therapy (ART) is the most important factor in the management of human immunodeficiency virus (HIV). This study examines the level of nonadherence to ART and the predictors of interventions to improve ART adherence in Jharkhand, a state with a predominance of tribes.

Methods: The present study was conducted at the ART center, Rajendra Institute of Medical Sciences (RIMS), Ranchi, Jharkhand. As it represents most patients from the state and was easily accessible to us, this center was taken as the study site. Considering ART intake, 30 tribal and 30 non-tribal patients were selected. All patients' baseline data was taken from the center and follow-up visits were done at their homes. Information regarding sociodemographic profile, ART regimen, adherence to ART, and reasons for nonadherence was collected. The interviews were recorded on audiotape, transcribed, and translated verbatim. We used a thematic approach to analyze the data.

Results: In our study, the prevalence of ART at the RIMS ART center was 21.7%, and the causes of non-adherence were asked in depth. The most common reason for nonadherence to treatment was the traveling distance and cost incurred in travel.

Conclusion: Comprehensive research can provide valuable insights into the reasons behind nonadherence and help develop targeted interventions to promote adherence. Some incentives can be provided along with treatment to cover long-distance travel expenses and loss of wages. Delivery of drugs to ART centers can be more regularized.

## Introduction

Today health is the priority issue to assure perceptible economic and social development in the fast-growing society. Health and the economy have been closely intertwined together. A robust and capable workforce enhances national prosperity by boosting overall productivity, fostering positive economic outcomes, and supporting the government and the country's thriving development. Human Immunodeficiency Virus (HIV) and Acquired Immuno Deficiency Syndrome (AIDS) pose a significant threat to a productive workforce. Individuals affected by HIV are often forced to confront a dual challenge: the risk of losing their income and livelihood, coupled with the experience of discrimination. According to the India HIV Estimates 2021, at the national level, the estimated adult HIV prevalence (15-49 years) has shown a decline since the peak of the epidemic in 2000 [1]. Prevalence was estimated at 0.55% in 2000, decreased to 0.32% in 2010, and further to 0.21% in 2021 [1]. The north-eastern region states exhibit the highest adult HIV prevalence, with 2.70% in Mizoram, 1.36% in Nagaland, and 1.05% in Manipur [2]. This is followed by the southern states, with 0.67% in Andhra Pradesh, 0.47% in Telangana, and 0.46% in Karnataka [2]. The estimated number of people living with HIV (PLHIV) stands at around 39 million all over the world with 6.5 million in the Asia Pacific region [3]. India ranks second in the world for its HIV epidemic, accounting for roughly 6.3% of all PLHIV globally [3]. In 2022, India is estimated to have approximately 24.67 lakh PLHIV, with an adult (15-49 years) prevalence of 0.20% [4].

The universality of the use of antiretroviral therapy (ART) and its easy availability and affordability has increased the survival of PLHIV still it is equally important to keep the viral load down and suppressed to ensure that the transmission of diseases is halted. According to UNAIDS globally, 28.7 million people living with HIV were receiving ART so its coverage was 75% (66-85%) in 2021 [3]. India’s share in PLHIV globally had come down to 6.3% (from around 10% two decades ago). As of the end of 2023, of all PLHIV, an estimated 82% knew their HIV status, 72% were on ART and 68% were virally suppressed [5].

The fight against the HIV/AIDS epidemic owes much of its progress to advancements in prevention and treatment. However, despite significant strides in scientific understanding, prevention, and treatment of HIV, along with extensive efforts by the global health community and governments, many individuals with HIV still lack access to prevention, care, and treatment, and a cure remains elusive. Now ART is recommended for all PLHIV irrespective of their CD4 cell count in order to consistently suppress the viral load, maintain elevated CD4 cell counts, prolong survival, and reduce the risk of HIV transmission to others [6].

Failure to adhere to ART can lead to serious consequences such as drug addiction, mental illness, and the transmission of infectious diseases like TB and hepatitis. Adherence, as defined by the World Health Organization (WHO), refers to the extent to which a patient can adhere to a treatment schedule and take medication at the recommended times [7]. In the context of HIV, failure to adhere to medication can result in viral rebound, potentially leading to immunosuppression and the development of viral resistance [7,8].

Research indicates that the effectiveness of ART hinges on how faithfully a patient follows the prescribed treatment regimen, dosages, timing, and other related instructions [9]. Studies suggest that for ART to achieve its maximum benefits, adherence rates exceeding 95% are recommended. Moreover, nonadherence to ART has been linked to the emergence of HIV drug resistance [6,10].

In countries like India, where there is a high prevalence of HIV/AIDS cases and significant socioeconomic and regional diversity, adherence to ART becomes particularly crucial. Many PLHIV are uneducated and have low awareness regarding the importance of maintaining ART.

Different studies point out that the factors influencing ART adherence like long distances to the center, travel expenses, ignorance of complications related to nonadherence and ART-related side effects, and non-acceptance and disclosure of HIV status demotivate the patients’ from following treatment [11,12]. Regular monitoring of adherence is vital to optimize drug effectiveness and achieve successful viral suppression.

Assessing the factors influencing adherence is crucial for enhancing treatment effectiveness and improving outcomes for patients on ART [9,13]. In resource-limited settings, adherence assessment has relied on records maintained by ART center staff due to its practicality. This research aims to assess the significance of patient and treatment factors in influencing nonadherence behavior. Additionally, there has been limited investigation into adherence among marginalized communities such as tribal groups. Therefore, the objective of our study was to identify in detail the factors influencing adherence levels of ART regimens among patients receiving care at an ART center.

## Materials and methods

Study design

It was an analytical-qualitative study. There are 13 ART centers in Jharkhand state. One of the centers located in the state capital, Ranchi was selected as our study site as it represents patients from all over the state. From this center, a list of tribal and non-tribal registered nonadherent patients was taken. Our study population was all adult people living with HIV registered at ART centers who had taken a minimum of six months of treatment. We selected a cohort of 60 patients who were planned to be interviewed. The information saturation principle was used and interviews were done till a complete response was obtained, and conducting further interviews gave no additional information so we conducted 32 interviews [14]. To get an overview of the program and find deficiencies in the service delivery, the counselor of the ART center was also interviewed. The study was done for 10 months from June 22 to March 23.

Inclusion Criteria

Adults registered at the ART center who had taken at least six months of treatment and gave proper written informed consent for interview.

Exclusion Criteria

Morbidly sick patients, those not available for the interview, and those not giving consent.

Plan of Study

We conducted a qualitative study, to find the perspectives of PLHIV regarding the factors influencing their ART adherence. The data for the last year was looked at to calculate the level of nonadherence to treatment. After finding the level of adherence, we tried to investigate the causes of nonadherence in detail. For this, tribal and non-tribal patients who were nonadherent to the treatment were selected. However, in interviews during the field visit, we observed that some reported nonadherent patients were taking regular medication from other sources. So, some adherent patients who were reported nonadherent were also included in the study. Each patient was interviewed personally using a semi-structured open-ended questionnaire. A total of 32 patients were interviewed. Counselors and pharmacists in the selected center were also interviewed in depth.

Operational definition

Adherent was defined as patients consuming ≥95% of the drug prescribed in 28 days time period (in the last six months), while nonadherent were patients who didn’t consume at least 95% of the drug prescribed in 28 days (during the last six months) due to any cause [10].

Data collection tools

Social science research assistants were recruited for the study, and trained on the study protocol and interview tools. The assistants conducted interviews with individuals living with HIV in their homes, ensuring privacy, and using the local language. Each interview lasted approximately 90-120 minutes. Interviewers prioritized building trust with the respondents to encourage openness about their experiences. Questions were posed while paying attention to participants' body language. Data were collected using an interview guide, and interviews were audio-recorded with additional field notes taken. The language used for interviews was either Hindi or local tribal dialect, based on the respondents' comprehension.

Data management and analysis

Data was entered in Micosoft Excel (Microsoft Corp., Redmond, WA, USA) and templates were generated. Mean and standard deviation were calculated for continuous data. Proportion was used for nominal and ordinal data. Appropriate statistical tests were applied, based on qualitative and quantitative data. The qualitative data was recorded in audio recorders during interviews and was transcribed and translated into English. The recorded transcripts underwent multiple readings to ensure thorough familiarization with the data [15]. Subsequently, manual coding was employed, and the codes were merged based on consensus among the authors. Finally, the coded data were summarized into overarching themes. Thematic analysis was conducted by identifying and analyzing major themes derived from the responses gathered during the interviews.

Ethics approval and consent to participate

All the necessary approvals were obtained from relevant institutions, and all methods adhered to applicable guidelines. The study was conducted after the approval of the Institutional Ethics Committee, RIMS, Ranchi with the approval number 65, dated May 21, 2022. Additionally, written informed consent, by the Declaration of Helsinki, was taken from all the study participants for the interview, audio recording, and publication ensuring anonymized responses.

## Results

The majority of patients were in a younger age group of 29-39 years (40%), with almost all (90%) being in the <60 kg category. More than 90% of patients were from rural backgrounds and 60% of patients were practicing Hindus, followed by Christians (20%). As we have preferably taken more tribal patients, they formed almost half of the total number, followed by (21%) BC (Backward Class) I (35%), and the general category (12%). The majority of patients (70%) were at least literate or had studied till the 10th. The major occupation was farming (30%) and daily wagers (18.33%) while (20%) were not working anywhere. The majority of participants were married (91%) and came from a nuclear family (71%). Almost half (46.7%) of the respondents belonged to the modified BG Prasad socioeconomic class IV [16], followed by (36%) SES (socioeconomic status) class V (Table 1).

**Table 1:** Sociodemographic characteristics of patients selected for interview SES: socioeconomic status; BC: Backward Class; SC: Scheduled class, ST: Scheduled Tribe

S. No.	Sociodemographic characteristics variable	Values	Total frequency
1.	Age in years	18-28 yrs	17 (28.33%)
		29-39 yrs	24 (40%)
		40-50 yrs	13 (21.67%)
		51-61 yrs	6 (10%)
2.	Weight in kilogram	<60	54 (90%)
		>60	6 (10%)
3.	Religion	Hindu	37 (61.67%)
		Muslim	2 (3.33%)
		Christian	11 (18.33%)
		Sarna	10 (16.67%)
4.	Residence	Urban	5 (8.33%)
		Rural	55 (91.67%)
5.	Category	General	7 (11.67%)
		BC-I	21 (35%)
		SC	3 (5%)
		ST	28 (46.67%)
		BC-II	1 (1.67%)
6.	Educational status	Illiterate	9 (15%)
		Just literate	10 (16.67%)
		Primary	16 (26.67%)
		Secondary	18 (30%)
		Higher secondary	6 (10%)
		Graduate and above	1 (1.67%)
7.	Occupation	Private job	4 (6.67%)
		Farming	18 (30%)
		Self-employed	2 (3.33%)
		Daily wages	11 (18.33%)
		Student	1 (1.67%)
		Unemployed	12 (20%)
		Driver	5 (8.33%)
		Others	7 (11.67%)
8.	Marital status	Married	55 (91.67%)
		Unmarried	5 (8.33%)
9.	Type of family	Nuclear	43 (71.67%)
		Joint	17 (28.33%)
10.	SES	Class I	1 (1.67%)
		Class II	2 (3.33%)
		Class III	7 (11.67%)
		Class IV	28 (46.67%)
		Class V	22 (36.67%)
	Total		60 (100%)

ART adherence among HIV-positive clients

The level of ART adherence among PLHIV, which is around 117 (78%), was calculated from the list of patients registered in the ART center in the last year. From this list, we interviewed 32 patients, after which a saturation in response was reached.

**Table 2:** Reasons for not taking ART treatment DOTS: directly observed treatment, short-course; ART: antiretroviral therapy

Predictors/reasons		Frequency	Percent
1	Ignorance/forgetting	1	3.0
2	Felt better and left	3	9.1
3	Long traveling distance to DOTS center	5	15.2
4	Travel expense	12	36.3
5	Social stigma	3	9.0
6	Engaged in social activities	2	6.1
7	Too busy with other things	7	21.3
	Total	33	100.0

Table 2 shows the overall main reasons pointed out by patients when asked why they were not taking ART treatment, they were further asked to elaborate on the situation they faced and what led them to drop out from treatment in in-depth interviews.

The study revealed important findings among HIV-positive patients regarding HIV infection, ART adherence, and their experience while taking drugs as well as factors that prevented them from continuing treatment (Table 3).

**Table 3:** Themes and sub-themes of nonadherence factors studied K/W: knowledge

Themes	Subthemes
Individual factors	K/W of HIV, source of infection, other work is given more priority, fear of side effects
Sociocultural factors	Stigma of disease, general ignorance, patient’s trust in health workers, more faith in traditional healers
Structural issues	Irregular supply of drugs, high transport cost, COVID-19 infection scare

Origin of disease

Most of the participants did not have any sign or symptom of the disease at the time of diagnosis. The disease was diagnosed incidentally when the patient came for treatment of any other disease. When asked about how the patients acquired the disease, most of them feigned ignorance and were unable to give a correct answer.

“I went for many treatments while in the army, could have acquired that way but can’t say clearly” (Male P03) (Table 4).

**Table 4:** Patient description of factors responsible for nonadherence to ART therapy K/W: knowledge, ART: antiretroviral therapy, RIMS: Rajendra Institute of Medical Sciences, NGO: non-governmental organization

Emerging themes	Selected statements of patients
Individual factors: K/W of HIV infection routes. K/W of benefits of taking ART regularly. Other work given more priority. Fear of side effects.	“I don’t know how I got this dreadful disease, I was not exposed in any manner” (F P05). “I went for many treatments while in the army, could have acquired that way but can’t say clearly” (M P03). “I was explained about regularity in consuming ART, earlier I was very regular in my treatment but it was interrupted during COVID-19, and since then not taken it” (F P16). “I know taking treatment is necessary but I have to look after other works too, sometimes I miss it” (M P27). “Earlier I used to take medicine, but somehow it was left due to family problems/financial condition, but I am still okay!” (M P09). “After taking ART drug I felt dizziness, I was restless and had weakness the whole way home” (F P19). “Whenever my wife took medicine she started having *daura* (traditional healer) used to be mentally disturbed the whole day” (F P23). “On taking medicine I felt weakness and was not able to even walk properly” (M P24).
Sociocultural factors: Stigma of disease. Patient’s trust in health worker. General ignorance. More faith in traditional healers.	"I am scared that if I am seen at the ART center any acquaintance can disclose the HIV status to other members of the community" (Female P05). “RIMS is a big hospital, and many people from my area go there, if I am seen at the ART center then, everyone will get to know I am living with HIV positive status” (M P28). “Sometimes people point at us saying that we are taking HIV medication, stay away from them and this is sad" (F P11). “When I started ART treatment, I did not want to continue because I was given only 15 days of drugs, when I asked for more the drug dispenser shouted at me in the presence of my daughter. I left taking drugs for months” (F P11). “Why should we take ART drugs anymore when I have no symptoms? I am cured now I don’t need drugs” (M P13). “Yes, medicine was given to me, I even ate it many days, I was not able to go recently but I will go again to take it” (F P08). “These drugs are not able to completely cure the disease, our *guniya* (traditional healer) promised me total recovery” (F P05). “English drugs are very hot we trust desi medicine more they suit us” (M P32).
Structural issues: Irregular supply of drugs. High transport cost. COVID-19 infection scare.	"When I started ART treatment, I used to get a monthly dose but now I am given only 15 days of drugs, sometimes just a week when I ask for more drugs, they say it is less in quantity” (F P11). “Due to irregularity in the flow of medicine, we give for a shorter duration (15 days) and many patients still came at the usual time ie one month for the next dose resulting in inadequate drug intake” (Pharmacist). “We can’t always go to the hospital every month to get medicine as it is quite far away, medicine is free but traveling only costs about Rs 250-400” (Male P01). “The day I go for medicine taking I have to be absent from my work, there are no earnings that day, how will our *chulha* (coal or wood-based stove) burn and food be cooked” (Male P07). “This was a problem for us as no transport vehicle was available and hiring an auto was very costly” (M P12). “It’s difficult to contact patients following irregular treatment as their numbers change or are switched off, and many are from difficult-to-reach areas,” “The traveling expenses and time taken to reach patients out of Ranchi district in far-flung areas is a lot, affecting our daily work,” “In time we reach an interior and difficult-to-reach area we can cover 3-4 nearby nonadherent patients, so we just leave them as such” (NGO workers following the irregular patients). “We have to take many precautions when going to hospital sometimes not having a mask was a challenge” (Female P08). “Hospital has lots of patients, also those of COVID-19, going there is equivalent to inviting infection” (F P17).

Healthcare worker-patient interaction

The majority of the participants expressed conflicted feelings toward treatment by health workers. The majority of them said they got along well with the health workers when visiting for drug taking while others said they were shouted at times. Some reported that they didn’t get full months of medicine and were scolded when asked more.

“When I started ART treatment, I did not want to continue because I was given only 15 days of drugs, when I asked for more the drug dispenser shouted at me in the presence of my daughter. I left taking drugs for months” (Female P11).

Considering transport cost

Most of the people reported that sticking to ART treatment was beneficial. However most of the participants not taking medication were doing so due to travel expenses and loss of wages on the day of traveling to ART center to receive drugs. Some of the views have been shared as quoted below.

“We can’t always go to the hospital every month to get medicine as it is quite far away, medicine is free but traveling only costs about Rs 250-400” (Male P01).

Effect of COVID-19 on compliance

Some people had stated difficulty in going to the hospital because of the COVID-19 lockdown as public transport was not available and they were scared of the hospital which was a COVID-19 treatment center. Some of the respondents stopped taking ART drugs during the peak of the COVID-19 pandemic and continued the same behavior even later.

“This was a problem for us as no transport vehicle was available and hiring an auto was very costly also we could go with precautions, sometimes not having a mask was a challenge” (Female P08).

General apathy and ignorance

The majority of the respondents had not taken medication for the last year because they were busy and did not get time to come to the ART center. Once they had a huge gap in treatment, they were not so motivated to restart the treatment as they remained asymptomatic.

“Earlier I used to take medicine, but somehow it was left due to family problems/financial condition… but I am still okay!” (Male P09).

Some respondents were not taking medication because their symptoms improved and they thought they did not have the disease anymore.

“Why should we take ART drugs anymore when I have no symptoms? I am cured now I don’t need drugs” (Male P23).

Stigma

Some patients in the group pointed out that visiting ART centers leads to stigma. Some participants reported that "if seen at ART center any acquaintance can share the HIV status with other members of the community" (Female P05).

“Sometimes people point at us saying that we are taking HIV medication, stay away from them and this is sad” (Female P21).

Other reasons

A few respondents were not taking medication due to side effects of the ART drugs like dizziness, weakness, or mental restlessness on taking medicine. But their number was very low (1-2).

Many patients complained about the long waiting time to receive the drugs from the ART center, leading to the loss of a whole day of work and wages.

Health workers' perspective

Pharmacists and other staff of the National AIDS Control Organization (NACO) reported irregularity in the flow of medicine so they had to give medicine for a shorter duration (15 days) and many patients still came at the usual time ie one month for the next dose resulting in inadequate drug intake. NACO staff and volunteers of NGOs professed the difficulty faced in contacting patients following irregular treatment as many were from difficult-to-reach areas and traveling expenses and time taken to reach them were a lot affecting their daily work.

## Discussion

There has been progress in managing HIV in India with increased access to ART being a significant factor in improving the prognosis for HIV patients globally. The work of organizations like the NACO is crucial in providing support and care to those diagnosed with HIV. While a complete cure for HIV remains elusive, advancements in medical care have made it possible for individuals living with HIV to lead longer and healthier lives. Continued efforts in education, prevention, and treatment are essential in further stabilizing the HIV situation and reducing its impact on individuals and communities. In our study, we tried to find the prevalence of ART drug adherence among the PLHIV, the level of nonadherence, and the reasons for the same. We observed a high ART prevalence of 78% compared to a systematic review done in India which showed an average prevalence of 70% adherence [17].

The open-ended approach of qualitative design allowed us to employ an individual experience-centered method to delve into the viewpoints of individuals living with HIV/AIDS, yielding comprehensive data to uncover gaps missed by alternative research methods [18,19].

We explored the barriers that influence PLHIV adherence to antiretroviral therapy in Jharkhand. Economic constraints and long travel time were recognized as significant barriers, stigma towards HIV with fear of disclosure of HIV status, lack of social support, general ignorance and forgetfulness, drug side effects, and COVID-19 lockdown restrictions also affected retention to therapy to a great extent. In coherence with our research, other studies reported distance and associated cost as one of the prime factors in being nonadherent to treatment [20,21]. Another study done in India showed a paradoxical observation with some patients preferring long distances from the ART center (to maintain confidentiality), few others expressed it as a detrimental factor in taking treatment [22]. Fear of disclosure of HIV status and social stigma and discrimination were also recognized as barriers in other studies done in America [23], Middle East [24], and Asia [25]. In our study ignorance and forgetfulness were also one of the reasons to drop ART treatment in chronic patients, which is consistent with the findings of other qualitative studies [26]. Adverse drug effects following ART consumption, congestion, and extended waiting periods at drug centers found as reasons for noncompliance were echoed in additional research conducted elsewhere and in India [20-22].

To improve compliance, we can follow further studies done in resource-limited settings like ours and form community groups, involving them in the administration process of ART which can lead to better adherence to treatment [27]. Moreover, our investigation revealed that people living with HIV (PLHIV) expressed fear of potential social ostracism from their families, friends, and the broader community, largely due to a lack of understanding among the public about virus transmission. Consequently, there is a pressing need for increased public education campaigns.

Limitations

This is a cross-sectional study; we have not followed up with the patients to observe medicine intake over an extended period of time. We have not collected data from bedridden or seriously ill patients. Also, this study was done at a single center, which limits the generalizability of the findings. So, the results should be interpreted with caution. Despite limitations, we hope the results of this study will provide additional evidence on the state of HIV care providing first-line insights into barriers, facilitators, perception of risk, and opportunities for enhancing adherence to PLHIV in developing countries like India.

## Conclusions

For any HIV care program to work properly regularity and adherence to ART must be focused. So, we interviewed 32 patients reported to be non-adherent to ART drugs for our study. As the majority cited the reasons for not taking ART as being long traveling distance, travel expenses, or loss of wages for attending the ART center, we recommend increasing the number of ART and linked ART centers so that centers are nearby decreasing the traveling distance and cost or should have provision to give drugs for a longer duration (three months) for patients coming from distant areas. Some reminders like phone calls/SMS or home visits for non-compliant patients can be started. Patients can be promoted to form community groups to support each other. NACO should ensure that there is a regular inflow of medicines and counseling services should be strengthened further.
